# Noninvasive Approach of *Plasmodium falciparum* Molecular Detection for Malaria Surveillance in Malaria Endemic Areas in Cameroon

**DOI:** 10.1155/2022/3600354

**Published:** 2022-11-09

**Authors:** Palmer Masumbe Netongo, Severin Donald Kamdem, Ardin Noutong Ouayoue, Theophile Patrick Djiokeng, Sophie Wankio Toju, Eric Berenger Tchoupe, Prisca Djivida, Jean Paul Chedjou, Irenée Domkam, Wilfred Mbacham

**Affiliations:** ^1^Molecular Diagnostics Research Group, Biotechnology Centre-University of Yaounde I, Cameroon; ^2^Department of Biochemistry, Faculty of Science, University of Yaounde I, Cameroon; ^3^Laboratory for Public Health Research Biotechnologies, Biotechnology Centre, University of Yaounde I, Cameroon; ^4^School of Science, Navajo Technical University, Crownpoint, New Mexico, USA; ^5^School of Health Sciences, Catholic University of Central Africa, Yaoundé, Cameroon; ^6^Centre de Reference International Chantal Biya, Yaoundé, Cameroon

## Abstract

**Background:**

Accurate, cost-effective, and noninvasive alternative molecular methods are needed for detecting low malaria parasitemia. The currently-used nested polymerase chain reaction (nPCR) requires blood as well as skilled personnel in order to minimise the risk of bloodborne disease transmission. Therefore, this study is aimed at assessing the accuracy of a noninvasive and more affordable malaria diagnosis with saliva using the loop-mediated isothermal amplification (LAMP) technique.

**Methods:**

A cross-sectional study was conducted in the Centre and Southwest regions of Cameroon. Matched blood and saliva samples collected from symptomatic and asymptomatic participants were tested for malaria using rapid diagnostic tests, microscopy, PCR, and LAMP. Statistics were performed using R studio software at 95% confidence interval.

**Results:**

A total of 100 participants (65% symptomatic and 35% asymptomatic) aged between 1 and 74 years with a balanced gender distribution ratio of 1.08 were included in our study. The prevalence of malaria was 61%, 57%, 59%, 42%, 35%, 17%, and 16% for blood-RDT, blood-PCR, blood-LAMP, blood-RT-LAMP, saliva-PCR, saliva-RT-LAMP, and saliva-LAMP, respectively. Both saliva and blood showed a sensitivity of 43.90% and respective specificities of 68.75% and 57.62%. When using RT-LAMP, sensitivities of 49.38% and 48.21% and specificities of 94.11% and 66.67% were recorded for saliva and blood, respectively. Sensitivities of 70.23% and 73.49% and specificities of 62.5% and 76.47% were recorded, respectively, for saliva-LAMP and saliva-RT-LAMP when compared to saliva-PCR as the gold standard. Saliva-LAMP and saliva-RT-LAMP had a fair agreement (*к* = 0.221 and 0.352, respectively) with saliva-PCR. Homemade LAMP and RT-LAMP technologies match the WHO recommendations and after proper validation in a larger sample size, could serve for malaria diagnosis in developing countries.

## 1. Introduction

Malaria remains a life-threatening disease caused by *Plasmodium spp*. and transmitted to humans by the female *Anopheles* mosquito [1]. Among the five human malaria species, *Plasmodium falciparum* is the deadliest[2]. Despite all the progress in combatting the malaria, the African continent continues to bear the highest burden, wherein around 90% of global deaths linked to the disease occur [3]. About 214 million cases and 409,000 deaths were still recorded worldwide [4]. In Cameroon as well as in other African sub-Saharan countries, malaria is the primary cause of morbidity and mortality, particularly in children below five years [5, 6].

Nowadays, several policies are being implemented to strengthen malaria control and elimination. Such approaches are primarily based on an accurate and prompt diagnosis, measuring the impact of any intervention, as well as using this as a prerequisite for effective treatment [7]. An accurate diagnosis of malaria is vital to avoid unnecessary presumptive treatment. Malaria diagnosis using clinical signs is most commonly practiced, but its accuracy varies widely as malaria symptoms overlap with many other tropical diseases. Thus, a practical, accurate, and reliable parasitological diagnosis is needed in all cases [8]. The use of microscopy on Giemsa-stained blood smears remains the gold standard for malaria diagnosis as it offers the advantages of quantifying the parasite density and the differentiation of *Plasmodium* species [8]. However, this diagnostic approach requires extensive training sessions and highly experienced personnel.

In remote settings without access to microscopy, malaria rapid diagnostic tests (RDTs) are frequently used to diagnose the disease. These cassette-based tests detect the *Plasmodium falciparum*–histidine-rich protein 2 (PfHRP2) and the parasite-specific lactate dehydrogenase (pLDH) proteins [9, 10]. However, the persistence of the PfHRP2 in the blood even after parasite clearance compromises the specificity (false positives) of this diagnosis technique [11]. Another concern is the recent discovery that up to 40% of *P. falciparum* parasites in Southern America harbored an HRP2 gene deletion, thus leading to false-negative results [12]. Considered the most sensitive technique, nested polymerase chain reaction (nPCR), using the *Plasmodium* genus-specific primers for the first amplification round and a species-specific primer for the second amplification, can detect parasitemia as low as 1–5 parasites/mL [13, 14]. Although this method is considered the “gold standard” due to its high sensitivity and specificity, it is arduous, expensive, and requires advanced skills and equipment thereby making it not suitable for a routine diagnosis[15].

Loop-mediated isothermal amplification (LAMP) is comparatively a simple and field-adaptable molecular technique [16] that allows parasite DNA to be amplified under isothermal conditions using *Bacillus stearothermophilus* (Bst) polymerase. From just a few copies of DNA, a 60-minute amplification through this method yields a precipitate of magnesium pyrophosphate in a positive reaction with high efficiency [16]. However, the invasive nature of blood-based tests requires the personnel to be well trained in order to reduce accidental transmission of bloodborne pathogens. The pain experienced by patients during blood collection impairs their willingness to participate in large-scale malaria surveillance programs [17]. To circumvent these challenges, many scientists are investigating the potential detection of *Plasmodium* DNA in noninvasive fluids such as urine and saliva as an alternative [18].

To develop a noninvasive strategy for malaria surveillance in endemic and resource-limited areas, this study is aimed at evaluating the sensitivity of the molecular diagnosis of *Plasmodium falciparum* using LAMP technology in saliva compared to blood.

## 2. Methods

### 2.1. Study Design and Areas

We conducted a cross-sectional study involving participants at Obala and Kumba district hospitals. The city of Obala is located in the Lekié division within the Centre Region of Cameroon. It has a population of 125 000 inhabitants and its surface area is 456 km^2^. Kumba is the largest city in the South-West Region with a population of 265 072 inhabitants and stands as the economic capital of the region.

The inhabitants of both cities are at high risk for malaria, and the disease was responsible for up to 28% of all consultations in 2012 in both towns. However, the National Malaria Control Program (NMCP) recorded that an alarming number of government-subsidized ACTs (~11000 doses) were sold in Kumba in 2012. This represents about 50% of the total number of ACTs sold in Yaounde during the same year (Challenges of ACT Subsidy: The Cameroon Case File).

### 2.2. Sample Size and Study Population

The study involved both symptomatic and asymptomatic participants, which is an approach generally used during disease surveillance. The sample population included outpatients sent for a malaria blood test (symptomatic participants) and people whose activities were carried out within our intervention area (asymptomatic participants). Throughout a period of 4 weeks, blood and saliva samples were collected from 65 symptomatic and 35 asymptomatic participants.

### 2.3. Sample Collection and Rapid Diagnostic Testing

We collected two 2 mL of venous blood by venipuncture from each consenting participant into an EDTA anticoagulant tube. Subsequently, a drop of blood was spotted on a labeled filter paper (Whatman®, Sigma-Aldrich, Germany) and dried for long-term storage in brown envelopes containing a desiccant. Finally, these envelopes were placed in an airtight plastic container and preserved in secured metallic trunks.

Saliva was collected from each participant using OMNIgene® ORAL (OM-501) kits (Genotek, Ottawa, Canada). For children who could not provide saliva, we used a sterile buccal swab to collect a sample of buccal fluid. Saliva samples in OMNIgene® ORAL kits were kept at ambient temperature until *Plasmodium* DNA was extracted. Saliva and blood samples were transported to the laboratory for molecular analysis,

### 2.4. Rapid Diagnostic Tests Using Blood

SD Bioline™ Malaria Ag Pf/Pan RDT cassette was used to perform rapid malaria diagnosis on all samples. Following the manufacturer's instructions, 5 *μ*L of EDTA blood was introduced into the small well of the cassette followed by the addition of 5 drops of buffer into the larger buffer well. The results were interpreted as positive when the control line plus either the Pf and Pan lines were visible (no matter the intensity of these lines). The RDT cassettes on which only the control line was seen were considered malaria negative. Results were considered invalid and the test was repeated when there was no visible control line on the SD Bioline™ Malaria Ag Pf/Pan RDT cassette. RDT-positive participants were referred to the clinician for adequate treatment following the national guidelines. Symptomatic participants with negative RDT testing were sent to the bacteriology laboratory for more investigations.

### 2.5. Molecular Assays

#### 2.5.1. DNA Extraction

DNA was extracted from dried blood spotted on Whatman paper, and saliva was stored in the OMNIgene® ORAL kit using Qiagen QIAmp Mini Kit DNA extraction kit (Cat No./ID: 51306, Germany). The extracted DNA was subsequently used for amplification by nested polymerase chain reaction (nPCR), loop-mediated isothermal DNA amplification (LAMP), and real-time LAMP (RT-LAMP).

#### 2.5.2. LAMP Assay

All LAMP reactions were performed as previously described by Port et al. [19]. in 2014. The reaction consisted of 1 *μ*L template DNA, 1 *μ*L of the loop primers mix at 25X (loop-B at 100 *μ*M and loop-F at 100 *μ*M), 1 *μ*L of the core primers mix at 25X (forward inner primer and backward inner primer at 100 *μ*M, F3, and B3 at 100 *μ*M), 5 *μ*L betaine (0.4 mmol/L), 12.5 *μ*L of the LAMP reaction buffer (100 mmol/L; KCl, 40 mmol/L; Tris-HCl (8.8 pH), 16 mmol/L; MgSO4, 20 mmol/L NH4SO4, 0.2% tween 20, deoxynucleotide triphosphates 25 mmol/L each), 1 *μ*L Bst DNA polymerase (8 U), 1 *μ*L hydroxynaphtol blue (120 mmol/L), and 2.5 *μ*L nuclease-free water to complete to a total reaction volume of 25 *μ*L. Reaction tubes were incubated for 45 minutes in a thermocycler at 60°C for DNA amplification, followed by a 2-minute incubation at 80°C for enzyme inactivation.

*(1) Endpoint Assessment*. A positive test was revealed by turbidity of the reaction mixture (resulting from the precipitation of magnesium pyrophosphate as a by-product) as well as a change of color from violet to light sky blue. There was no color change in negative samples as the reaction persisted as violet.

#### 2.5.3. RT-LAMP Assays

The final reaction volume for real-time LAMP was 20 *μ*L and ran in the portable Genie II real-time fluorometer (OptiGene, UK). The reaction consisted of 12 *μ*L of 1X Isothermal Master Mix containing inorganic pyrophosphatase, Geobacillus DNA polymerase; buffer; MgSO4; dNTPs; ds-DNA binding dye (6-Carboxyfluorescein (FAM) detection channel); 3 *μ*L of core and loop primers mix (as described in LAMP procedure) and 3 *μ*L of the template DNA. All assays were run at 65°C for 60 minutes followed by a heating and cooling step to 98–80°C (0.05°C/s) to allow reannealing of amplified DNA and display of the amplification and the annealing curve. The Genie II displays amplification signals in real-time and at the end of the run, shows the time to positivity, the amplification, and annealing curves for each positive specimen.

### 2.6. Nested PCR

All 100 samples were tested for the genus *Plasmodium* and *P. falciparum* by nested PCR targeting the 18S rRNA gene. The first-round amplification targeting the *Plasmodium* species gene was conducted using specific genus primers rPLU 5 (5′-CCTGTTGTTGCCTTAAACTTC-3′) and rPLU 6 (5′-TTAAAATTGTTGCATTAAAACG-3′), whereas the second round which was specific to *P. falciparum* used rFal 1 (5′-TAAACTGGTTTGGGAAAACCAAATATATT-3′) and rFal 2 (5′-ACACAATGAACTCAATCATGACTACCCGTC-3′) primers. Both rounds of amplification reactions were performed in a total volume of 20 *μ*L using the OneTaq Hot Start Master Mix. For external reactions (genus-specific PCR), the extracted DNA was used as the template while 2 *μ*L of the purified external PCR products were used as the template for the internal reactions (species-specific PCR).

### 2.7. Outer PCR Reaction

The reaction mixture of the first PCR step consisted of 2 *μ*L of DNA template, 10 *μ*L of a 1X OneTaq Hot Start Master Mix (containing the KCl: 11 mmol/L; Tris-HCl: 10 mmol/L; NH_4_Cl_2_: 11 mmol/L; MgCl2: 0.9 mmol/L; and 0.1 mmol/L of each dNTPs, 25 units of Taq DNA polymerase); 0.5 *μ*L of a (10 × 10^−3^ mM) of each primer (rPLU 5 and rPLU 6) and water to a final volume of 20 *μ*L. Conditions for the primary amplification were as follows: initial denaturation of 95°C for 5 minutes; 25 cycles (denaturation at 94°C for 1 minute, annealing at 58°C for 2 minutes, and extension at 72°C for 2 minutes); and a 5-minute final extension at 72°C.

### 2.8. Inner PCR Reaction

Two microliters (2 *μ*L) of the first amplification product were used as DNA template for the second amplification. The conditions and concentrations of the second amplification were identical to those of the primary, except for the number of cycles (30 instead of 25). Also, 0.5 *μ*L of a (2.5 × 10^−5^ mM) rFal 1 and rFal 2 was used as primers.

The DNA extracted from the Pf_3D7 parasite strain was used as the positive control while nuclease-free water was used as the negative control. The PCR products from the second amplification round were analyzed by gel electrophoresis followed by ethidium bromide staining.

### 2.9. Agarose Gel Electrophoresis

The PCR amplicons were analyzed on a 2% agarose gel (Sigma, Fisher, USA) by electrophoresis at 100 V for 45-60 minutes, and the gel was later stained with ethidium bromide (Sigma, Aldrich, USA) to visualize the bands of the appropriate size on a UV transilluminator. LAMP and RT-LAMP amplicons were confirmed by 2% agarose gels. Molecular weight markers used as a reference were 100 bp DNA ladder (New England Biolabs, USA, Catalogue #: N3231L). Samples were considered positive if the amplicon with the anticipated size was revealed.

### 2.10. Quality Control

To validate PCR and LAMP results, negative control (master mix containing nuclease-free water) and positive control (3D7) were included in each series of amplification and gel electrophoresis. To confirm the homemade LAMP results, all reaction tubes were assessed by at least three independent researchers who were blinded to one another's results and RDT results as well as clinical data.

### 2.11. Ethical Considerations

Ethical clearance N° 2015/06/602/CE/CNERSH/SP was obtained from the National Ethics Committee on Research for Human Health (CNERSH) in Cameroon. Research authorisations were obtained from the Kumba and Obala District Hospitals and the Biotechnology Centre of Nkolbisson in Yaoundé. An informed consent/assent form was obtained from each participant or a legal representative.

### 2.12. Statistical Analysis

The results were analyzed with the R studio software (https://rstudio.com/) from which the sensitivities, specificities, and positive and negative predictive values were calculated. The Kappa test was performed to evaluate the agreement between techniques. Plots were generated using GraphPad Prism 8 (https://www.graphpad.com/). All statistics were performed at a threshold of 0.05.

## 3. Results

### 3.1. Sociodemographic Data

We collected specimens from 100 participants during the study period. Sociodemographic data of the study population are summarised in Table 1.

Of the 100 participants, 52% were female and 48% were male with a gender female/male ratio of 1.08. While 65% of participants were symptomatic, 35% were asymptomatic. Participants from rural areas were the most represented (64%) in our study population and the overall mean age was 24.74 years. Symptoms such as fever, headache, weakness, nausea/vomiting, and abdominal pain were recorded in 60%, 41%, 41%, 19%, and 19% of participants, respectively.

### 3.2. Malaria Prevalence by Gender, Age Group, and Techniques

**Figure 1:**
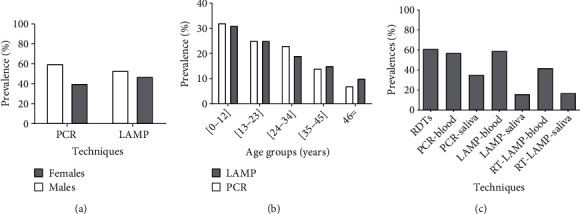
Malaria prevalence by gender and age group. (a). Malaria prevalence in male and female participants by PCR and LAMP. (b). Malaria prevalence in each age group by PCR and LAMP. (c). Malaria prevalence of different techniques using blood and saliva.

Males had a higher prevalence in the study population distributed as 59.65% by PCR and 52.55% by LAMP (Figure 1(a)).

The 0-12 years age group had the highest malaria prevalence by PCR (31.58%) and LAMP (30.50%). With a prevalence of 7.06% and 10.19%, respectively, by PCR and LAMP for the age group ranged 46 years and above, the occurrence sharply decreased with the increase in age (Figure 1(b)).

Using different techniques, malaria prevalence was 61%, 57%, 35%, 59%, 16%, 42%, and 17%, respectively, for RDT, blood-PCR, saliva-PCR, blood-LAMP, saliva-LAMP, blood-RT-LAMP, and saliva-RT-LAMP. Blood-LAMP detected 2 more malaria infections than blood-PCR, whereas saliva-LAMP detected 19 fewer malaria infections than saliva-PCR (Figure 1(c)).

### 3.3. Diagnostic Performance of the Different Methods with PCR as Standard

#### 3.3.1. Using Blood Samples

Blood-PCR was used as the standard to determine the performance of each method using blood as the sample. The diagnostic performances are summarised in Table 2.

Among molecular methods, when compared to blood-PCR, blood-LAMP had the lowest sensitivity and specificity with 43.90% and 57.62%, respectively, while RDTs had the highest sensitivity and specificity with 62.16% and 70.50%, respectively. However, although blood-LAMP had the lowest sensitivity, its observed agreement was higher than the expected agreement.

#### 3.3.2. Using Saliva Samples

To determine the performance of each method using saliva, saliva-PCR was used as the standard. The diagnostic performances are summarised in Table 3.

When compared to saliva-PCR, the sensitivities and the specificities of saliva-LAMP and saliva-RT-LAMP were 70.23%, 73.49%, 62.5%, and 76.47%, respectively, while the positive and negative predictive values were 90.76%, 93.84%, 28.57%, and 37.14%, respectively.

Saliva-LAMP and -RT-LAMP had a fair agreement (kappa 0.221 and 0.352, respectively) with saliva-PCR. But, in both cases, the observed agreements were higher than the expected agreement, which is described as follows: 69.00% against 60.20% for saliva-LAMP and 74.00% against 59.90% for RT-LAMP.

#### 3.3.3. Using Saliva versus Blood Samples

We compared saliva and blood samples using blood-PCR as the gold standard. In addition, since the goal of the study was to develop a noninvasive molecular method, saliva-LAMP was compared to blood-PCR (Table 4).

Saliva-PCR and saliva-LAMP showed sensitivities of 49.20% and 43.90%, respectively. The specificities of 71.42% and 68.75% were recorded for saliva-PCR and saliva-LAMP. Moreover, observed agreements were higher than expected, which is described as follows: 57.14% against 47.67% for saliva-PCR and 47.96% against 44.50% for saliva-LAMP (Table 4).

### 3.4. Electrophoresis as a Confirmation Method of the Amplification

#### 3.4.1. PCR

**Figure 2:**
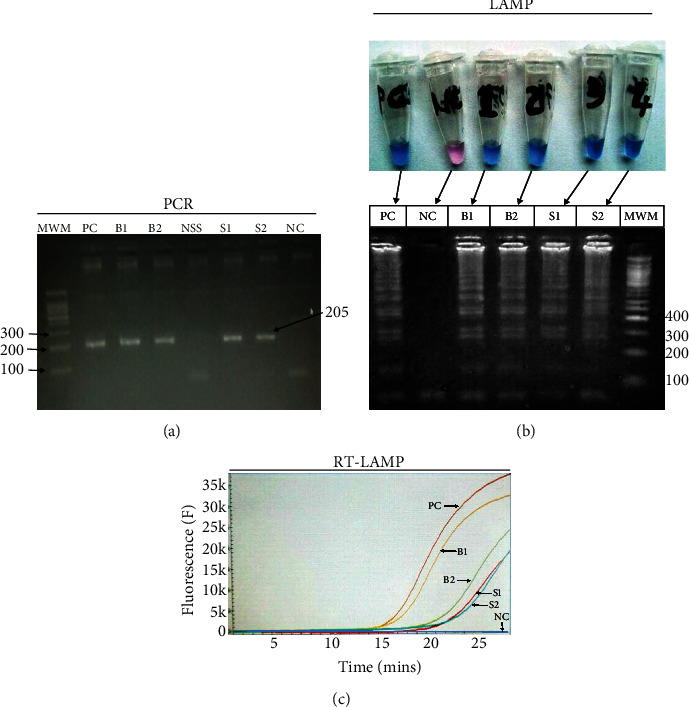
Electropherograms of *P. falciparum* in blood and saliva using PCR and LAMP. (a). Electropherogram of *P. falciparum* in blood and saliva using PCR. (b). Electropherograms of *P. falciparum* in blood and saliva using LAMP and the image of tubes showing the changing of color. (c). *P. falciparum* amplification curves by the real-time LAMP Genie II system. PC: positive control; B1: blood sample 1; B2: blood sample 2; NC: negative control; S1: saliva sample 1; S2: saliva sample 2; NSS: negative saliva sample; MWM: 100 base pair molecular weight maker represented on the gels by band size numbers only.

Of all the 100 participants diagnosed with *Plasmodium falciparum* by nested PCR, 57 were positive when blood was analyzed while 35 were positive with saliva. Electrophoresis of blood and saliva is shown in Figure 2(a). *Plasmodium falciparum* was identified as a DNA amplicon of molecular weight of about 205 bp for both saliva and blood (Figure 2(a)) [13].

#### 3.4.2. LAMP

From all the 100 participants tested for *Plasmodium falciparum* by LAMP, 59 were positive with blood and 16 with saliva. The gel and the color change from violet (negative reaction) to sky blue (positive reaction) of blood and saliva due to hydroxynaphthol blue are shown in Figure 2(b).

#### 3.4.3. Real-Time LAMP

From all the 100 participants tested for *P. falciparum* by real-time LAMP, 42 were positive with blood and 17 with saliva. The sigmoid amplification curves characterized the positive reactions whereas a straight line was considered a negative reaction (Figure 2(c)).

## 4. Discussion

Noninvasive, cheap, rapid, and accurate diagnostic methods could greatly enhance the success of malaria surveillance activities in very remote settings. The present study is aimed at determining the accuracy of the molecular detection of *Plasmodium falciparum (P. falciparum*) in saliva using LAMP technology when compared to blood. With one hundred (100) participants included in our study, our sample size is comparable to the study of Paris et al. in 2007 [20] and Najafabadia et al. in 2014 [21] with 115 and 108 participants, respectively. The age range trend was similar between our study and the two previously mentioned studies. Blood-PCR used in this study as the gold standard gave a malaria prevalence of 61% which is close to the prevalence obtained in the Bangladesh study conducted by Paris et al. in 2007 with 58.3%. This high prevalence could be explained as most of the participants were symptomatic (65.0%). When compared to blood-PCR, blood-LAMP had a sensitivity, specificity, positive predictive value, and negative predictive value of 43.90%, 57.62%, 41.86%, and 57.62%, respectively. These values are lower than those obtained by Najafabadia et al. in 2014 [21] and Kimbikuokuo et al. [22] with 95.8%, 100%, 100%, and 51.4%, respectively. These differences could be explained by the fact that our study was done on symptomatic and asymptomatic participants, while Najafabadia et al. only worked with febrile patients. Blood-RT-LAMP performance in terms of sensitivity, specificity, positive predictive value, and negative predictive value was higher than blood-LAMP with 48.21%, 66.07%, 65.85%, and 49.12%, respectively. These differences could be attributed to the subjective nature of the visual interpretation of LAMP results compared to the fluorometric measurements used in RT-LAMP.

Compared to saliva-PCR, saliva-LAMP and RT-LAMP showed an observed agreement higher than the expected agreement, respectively, 69.00% versus 60.20% for saliva-LAMP and 74.00% versus 59.90% for RT-LAMP (kappa 0.221 and 0.352, respectively). With a PPV, respectively, of 90.76% and 93.84%, these techniques can be of fair use for clinicians as the PPV is the parameter that correlates patients' symptoms to the presence of the disease. However, compared to blood-PCR, saliva real-time LAMP showed 49.38% sensitivity and 94.11% specificity, whereas saliva-LAMP sensitivity and specificity were a bit lower (43.90% and 68.75%, respectively), although the PPV was 97.56% and 87.80%, respectively. Our LAMP sensitivity and specificity in saliva samples were lower than the values (47% and 100%) found by Najafabadia et al. in 2014 [21] but similar to Cuadros et al. [23], whereas real-time LAMP sensitivity and specificity were greater. The low sensitivities of saliva-LAMP and real-time LAMP compared to nested blood PCR assay could indicate that the parasite DNA load in saliva is probably lower than the detection limit of 30 ± 5 parasites/ml reported by Lu et al. in 2012 [24]. Real-time LAMP sensitivity was comparable to nested PCR assay in saliva samples (73.49%), but it was a bit lower in saliva-LAMP (70.23%). This may indicate that real-time LAMP is more sensitive than LAMP. In the LAMP assay, higher parasitemia may probably increase the parasite load in saliva samples. It might be a correlation between the parasite level in the blood and their transfer in saliva. Mfuh and collaborators in 2017 found that PCR on saliva collected in Canada's DNA Genotek Inc. Kits was able to detect 53.3% of all submicroscopic infections that could be detected by blood-PCR [25]. The two molecular methods have been shown in our current study to have a good agreement. When homemade or field-adapted LAMP on saliva samples was compared to standard saliva-PCR, the observed agreement was 69% versus an expected agreement of 60.2% (kappa = 0.221, standard error = 0.088, *Z* = 2.516, *P* value = 0.006). The sensitivity was 70.3%, with a positive predictive value of 90.8% while specificity was 62.5% with a negative predictive value of 28.5%. This means that no matter what molecular technique is employed, diagnosis using saliva leads to comparable results.

Although both assays showed low sensitivity for the diagnosis of *P. falciparum* in saliva when compared to nested PCR blood, they seem to be very good for malaria diagnosis in large-scale programs. Considering LAMP technology's field adaptability and performance, in regard to the ease of saliva sampling, warrants the need for further research with bigger sample sizes. The high performance of the real-time LAMP, as the results determination is done through an inexpensive (when compared to regular thermocyclers) fluorescent-based device, can more easily be implemented in resource-limited settings with acceptable results. Despite the fact that LAMP and real-time LAMP technologies in their current form match the WHO recommendations for an acceptable diagnostic test for developing countries, room for improvement is attainable. In fact, if the sensitivity of the LAMP assays can be improved by targeting the mitochondrial gene with a 30–100 copy number sequences per parasite [26, 27] compared to 4-8 copies for the 18 s rRNA gene [28], the chance of detecting small amounts of parasite DNA in saliva could also be improved. The simplicity, easy availability, and noninvasiveness of saliva sampling (that can be performed even by patients with limited training) ought to inspire further extensive studies, with a larger number of clinical samples and fine tuning of the protocols to improve its usefulness in diagnosis during malaria surveillances.

## Figures and Tables

**Table 1:** Characteristics of the study population.

	Asymptomatic (*n* = 35)	Symptomatic (*n* = 65)	Total (*n* = 100)
Age (years)			
Range	[4–67]	[1–74]	[1–74]
Mean	24.74	22.41	29.05
Gender			
Male (%)	13 (37.1)	35 (53.8)	48 (48)
Female (%)	22 (62.9)	30 (46.2)	52 (52)
Residence type			
Rural (%)	23 (65.7)	41 (63.1)	64 (64)
Urban (%)	12 (34.3)	24 (36.9)	36 (36)

**Table 2:** Diagnostic performance of the different methods with blood.

Test characteristics	Blood-LAMP	Blood-RT-LAMP	RDTs
Sensitivities (%)	43.90	48.21	62.16
Specificities (%)	57.62	66.67	70.50
PPV (%)	41.86	65.85	56.10
NPV (%)	57.62	49.12	75.44
Observed agreements (%)	52.00	56.12	67.35
Expected agreements (%)	51.26	48.83	52.00
Kappa values	0.015	0.142	0.340

PPV: positive predictive values; NPV: negative predictive values.

**Table 3:** Diagnostic performance of the different methods with saliva.

Test characteristic	Saliva-LAMP	Saliva-RT-LAMP
Sensitivities (%)	70.23	73.49
Specificities (%)	62.50	76.47
PPV (%)	90.76	93.84
NPV (%)	28.57	37.14
Observed agreements (%)	69.00	74.00
Expected agreements (%)	60.20	59.90
Kappa values	0.221	0.352

PPV: positive predictive values; NPV: negative predictive values.

**Table 4:** Diagnostic performance of saliva versus blood.

Test characteristic	Saliva-LAMP	Saliva-PCR
Sensitivities (%)	43.90	49.20
Specificities (%)	68.75	71.42
PPV (%)	87.80	75.60
NPV (%)	19.29	43.85
Observed agreements (%)	47.96	57.14
Expected agreements (%)	44.50	47.67
Kappa values	0.062	0.181

## Data Availability

Complete data used in this study is available from the corresponding author upon request.
